# Exploration of a panel of urine biomarkers of kidney disease in two paediatric cohorts with Type 1 diabetes mellitus of differing duration

**DOI:** 10.1186/s13098-022-00839-4

**Published:** 2022-05-12

**Authors:** Letizia Zeni, Anthony G. W. Norden, Elena Prandi, Carolina Canepa, Keith Burling, Katherine Simpson, Barbara Felappi, Alessandro Plebani, Giovanni Cancarini, Pietro Manuel Ferraro, Donald Fraser, Robert J. Unwin

**Affiliations:** 1 Nephrology Unit, Azienda Socio-Sanitaria Territoriale degli Spedali Civili di Brescia, Piazzale Spedali Civili, 1-25123 Brescia, Italy; 2 Department of Renal Medicine, Royal Free Hospital Trust, University College London, Rowland Hill Street, London, NW3 2PF UK; 3 Pediatrics Clinic, Department of Clinical and Experimental Sciences, University of Brescia and ASST Spedali Civili of Brescia, Piazzale Spedali Civili, 1-25123 Brescia, Italy; 4 Department of Nephrology, Royal Berkshire Hospital, Reading, UK; 5 Core Biochemical Assay Laboratory, Cambridge University Hospitals NHS Foundation Trust, Hills Road, Cambridge, CB2 0QQ UK; 6 Wales Kidney Research Unit, Division of Infection and Immunity, School of Medicine, College of Biomedical and Life Sciences, Cardiff University, Cardiff, UK; 7 Nephrology, Department of Medical and Surgical Specialties, Radiological Science and Public Health, University of Brescia, Brescia, Italy; 8 U.O.S. Terapia Conservativa della Malattia Renale Cronica, U.O.C. Nefrologia, Fondazione Policlinico Universitario A. Gemelli IRCCS, Largo Agostino Gemelli 8, 00168 Rome, Italy; 9 Università Cattolica del Sacro Cuore, Rome, Italy

**Keywords:** Diabetic kidney disease, Urinary biomarkers, Type 1 diabetes mellitus, Albuminuria, microRNAs, Urine free retinol-binding protein 4, Kidney injury molecule-1

## Abstract

**Background:**

The pathogenesis of diabetic kidney disease (DKD) is complex and involves both glomerular and tubular dysfunction. A global assessment of kidney function is necessary to stage DKD, a progressive kidney disease that is likely to begin in childhood. The present study evaluated whether kidney injury biomarkers identified as early DKD biomarkers in adults have any prognostic value in the very early stages of childhood diabetes.

**Methods:**

We measured urine free Retinol-binding protein 4 (UfRBP4), albumin (UAlb), Kidney injury molecule-1 (KIM-1) and the microRNAs miR-155, miR-126 and miR-29b in two cohorts of paediatric T1DM patients without evidence of DKD, but with diabetes of short-duration, ≤ 2.5 years (SD, n = 25) or of long-duration, ≥ 10 years (LD, n = 29); non-diabetic siblings (H, n = 26) were recruited as controls. A p value < 0.05 was considered significant for all results.

**Results:**

UfRBP4 and UAlb were not significantly different across the three groups. No differences were found in KIM-1 excretion between any of the three groups. UfRBP4 was correlated with UAlb in all three groups (r 0.49; p < 0.001), whereas KIM-1 showed no correlation with albumin excretion. Among microRNAs, miR-29b was higher in all diabetic children compared with the H control group (p = 0.03), whereas miR-155 and miR-126 were not significantly different. No differences were found between the SD and LD groups for all three microRNAs. No associations were identified between these biomarkers with sex, age, BMI, eGFR, T1DM duration or glycaemic control.

**Conclusions:**

UfRBP4, KIM-1, miR-155, and miR-126 were unaffected by the presence and duration of diabetes, whereas miR-29b showed a modest elevation in diabetics, regardless of duration. These data support the specificity of a panel of urine biomarkers as DKD biomarkers, rather than any relationship to diabetes per se or its duration, and not as early DKD biomarkers in a paediatric setting.

**Supplementary Information:**

The online version contains supplementary material available at 10.1186/s13098-022-00839-4.

## Introduction

Although complications such as diabetickKidney disease (DKD) in early onset Type1 diabetes mellitus (T1DM) in children are usually not detected clinically until reaching adulthood, the underlying pathology is likely to begin in childhood. Indeed, structural changes in the kidney have been reported in humans and animal models early in the course of diabetes mellitus (DM) [1, 2]. Moreover, a ‘non-albuminuric’ form of DKD is becoming a better accepted and recognised clinical phenotype [3]. Although glomerular pathology may be the earliest structural event in DKD, tubular injury is also involved at an early stage [4]. Therefore, biomarkers that reflect both glomerular and tubular injury may prove useful in the early detection of DKD. Moreover, a comprehensive kidney biomarker panel would ideally include both biomarkers of tubular function and structure/injury concurrently to overcome limitations of a single biomarker approach such as albuminuria [5].

The need for early and reliable biomarkers of DKD, other than microalbuminuria alone, is increasingly important, not only in screening for DKD and its earlier detection, but also as potential clinical trial endpoints following an intervention. Currently, patients with non-albuminuric DKD are largely excluded from early (Phase 2) clinical trials typically of 6–12 months duration, because albuminuria reduction is still the most widely used surrogate endpoint for new drug treatment in DKD clinical trials [6, 7].

The free form of Retinol-Binding Protein 4 (fRBP4) is a 21 kDa low molecular weight protein (LMWP) recognized as a functional biomarker of the proximal tubule (PT) in the setting of acute and chronic tubular dysfunction [8]. There is clinical evidence that the free form of RBP4 is a more sensitive marker of proximal renal tubular dysfunction than measurement of total RBP4, which is used currently [9]. Previous studies have shown elevated urinary RBP4 in patients with T1DM [10, 11].

Kidney injury molecule (KIM)-1 is an immunoglobulin superfamily protein that is markedly upregulated in the proximal tubule of the injured or diseased kidney, and its urinary levels have been reported to be higher earlier in patients with T1DM and progressive DKD [12, 13]. It is considered a structural biomarker that is expressed on the apical membrane of proximal tubule cells and may reflect the severity of tubular cell injury [14]. Recently, a relationship between KIM-1 expression and fibrosis-inflammation in DKD patients has been shown, making KIM-1 itself a potentially attractive therapeutic target in DKD [12].

Urinary microRNAs are another recently explored means of studying the kidney, reflecting structural, functional and pathophysiological pathways along the nephron. Using a profiling approach of 754 microRNAs in pooled urine samples of DKD patients, miR-155, miR-126 and miR-29b were significantly increased and this has been confirmed in an independent cohort of patients with DKD, with DM without DKD, and in controls [15]. Based on these reported findings, we included these three microRNAs in our study.

In view of the glomerular-tubular interplay in protein handling, the concept of DKD as a glomerulo-tubular disorder, and existing data supporting increased excretion of UfRBP4, KIM-1 and microRNAs in patients with longstanding DM, we designed the present study to explore several potential kidney injury biomarkers in children and young adults with different durations of T1DM.

Our principal aim was to determine whether a panel of biomarkers that includes UfRBP4 (glomerular and tubular disfunction), KIM-1 (an early and widely recognized marker of tubular injury and DKD progression) and the three microRNAs, miR-155, miR-126 and miR-29b, might prove useful early renal biomarkers in a normoalbuminuric cohort of young diabetic patients, and if there is any correlate with diabetic control and disease duration.

## Materials and methods

### Study design, patient selection and procedures

All subjects were recruited from the T1DM Outpatient Clinic at the Paediatric Service of Azienda Socio-Sanitaria Territoriale (ASST) Spedali Civili di Brescia. Healthy controls were siblings of the diabetic patients in the clinic. Recruitment began in July 2017 and was completed in June 2018. Specimens preserved on dry ice were sent to the Core Biochemical Assay Laboratory (CBAL) of Cambridge University Hospitals NHS Foundation Trust, UK to measure UfRBP4, Ualb and KIM-1 and to the Wales Kidney Research Unit (Cardiff, United Kingdom) to measure microRNAs.

Inclusion criteria for Short Duration (SD) T1DM patients were onset of T1DM ≤ 2.5 years; for the Long Duration (LD) T1DM group onset of T1DM ≥ 10 years. The SD group was selected to detect any very early change in urine biomarkers excretion as according to current guidelines, the screening for DKD in T1DM begins only after 5 years from diagnosis [16]. The cut off of ten years to classify adolescents as long-standing disease was considered a reasonable time period for adolescents still in paediatric follow-up, since childhood onset of T1DM has a bimodal distribution, with one peak at four to six years of age and a second in early puberty. Healthy controls (H) had no evidence of DM determined by random fasting blood glucose measurement over the six months before study. Exclusion criteria for all subjects included: paediatric chronic kidney disease (CKD)*,* albuminuria (albumin-to-creatinine ratio > 3 mg/mmol) [17], persistent haematuria on dipstick testing*,* congenital anomalies of the kidney and urinary tract (CAKUT)*,* chronic hypertension, inflammatory or neoplastic diseases, liver disease, recurrent urinary tract infection, presence of Type 2 DM, age < 6 months and > 19 years. Autoimmune thyroiditis or celiac disease were not exclusion criteria.

Urine for routine analysis was collected as a midstream second void in the morning and sent immediately to the local clinical biochemistry laboratory. Urine for measurement of Urine free Retinol-Binding Protein 4 (UfRBP4), urinary albumin (Ualb), KIM-1 and microRNAs was put on wet ice immediately and frozen at −80 °C within one hour. Diabetic patients were also tested for asymptomatic urinary tract infection by urine routine culture. Pre-analytical procedures were carried out with standardized protocols for all patients. This is crucial, especially considering the sensitivity of proteins to degradation and the unfavourable milieu of urine as a source of biological material. RBP in urine samples is unaffected by freezing at −70 °C for short-term storage [18] and KIM-1 is stable at −80 °C for up to 5 years [19]. MicroRNAs are relatively stable in urine under a variety of storage conditions and after multiple freeze–thaw cycles [20, 21].

For diabetic patients, after an overnight fast, measurements included glycated haemoglobin, blood glucose, triglycerides, total cholesterol, high density lipoprotein, antithyroid antibodies, anti-transglutaminase antibodies, thyroid-stimulating hormone, serum creatinine, urate, aspartate aminotransferase, alanine aminotransferase and gamma-glutamyl transferase.

Except for three patients, urine and blood tests were collected on the same day. Any potential confounders, such as fever, severe exercise and vaccinations were avoided during the three days before collection.

Serum creatinine was not measured in healthy controls because of ethical restrictions for venepuncture in healthy children.

BMI was calculated as weight (Kg) divided by height squared (m^2^). We used an online calculator that provides BMI, percentile and z-score [22] BMI was age- and sex-adjusted [23]. Blood pressure percentiles were calculated for each patient.

Guidelines from the American Diabetes Association (ADA) and the International Society for Paediatric and Adolescent Diabetes (ISPAD) specify a patient target of HbA1c < 59 mmol/mol and such patients are considered to have good glycaemic control [16].

T1DM-related complications were defined as any acute complications requiring admission to hospital.

The threshold for defining hyperfiltration is challenging and a generally accepted definition is lacking. Some authors report thresholds corresponding to a GFR that exceeds two standard deviations above the mean GFR in healthy individuals. Notably, the use of any GFR cut off does not consider differences between genders, ethnicity, nephron endowment at birth and age [24]. The Schwartz formula tends to overestimate the actual creatinine clearance and should be used with caution. Moreover, the application of the Schwartz formula is suggested from 1 to 16 years age range, although many authors tend to use this formula until 19 years old [25]. To compare SD and LD-groups, we arbitrarily decided to use the Schwartz equation in all patients regardless of the age. In the present study, hyperfiltration was defined as an eGFR that exceeds two standard deviations above mean GFR of an age-matched normal (i.e., without evidence of renal disease) population, where glomerular filtration rate was determined by inulin clearance [26].

### Measurement of UfRBP4, Ualb, KIM-1 and MicroRNAs

Urine creatinine was measured by the Siemens Dimension^®^ RxL enzymatic creatinine assay. Two minor modifications were made to the original UfRBP4 assay to improve assay performance at low analyte concentrations: sample pre-dilution was one in two instead of one in five and an additional low standard was added to the standard curve. The lower limit of detection (corrected for dilution) of UfRBP4 in the original urine was 1.0 µg/L. KIM-1 was measured by Quantikine^™^ Elisa (R&D Systems, MN, USA). MicroRNA analysis proceeded according to our previously developed protocol [27] RNA was isolated from all samples using the miRNeasy kit (Qiagen, Crawley, West Sussex, UK) with the following modifications to the manufacturer’s protocol. Carrier RNA (MS2 RNA; Roche Diagnostics Limited, Burgess Hill, West Sussex, UK) was added at 1 μg/750 μL of QIAzol reagent, and 0.5 pM *Caenorhabditis elegans* miR-39 (MC10956, cel-miR-39; Life Technologies, Paisley, Renfrewshire, UK) was spiked into each sample to act as an internal control. Reverse transcription (RT) was carried out using the High-Capacity cDNA RT Kit (4368814, Life Technologies, Carlsbad, MA, USA) before quantification of specific microRNAs using TaqMan microRNA assays (Life Technologies).

### Data analysis and statistical power

Continuous variables with a normal distribution were expressed as mean ± standard deviation (SD); variables with a non-normal distribution as median and interquartile range (quartile 1 and quartile 3). To test for normality, we used the D'Agostino-Pearson Test. ANOVA, Unpaired Student t test, Wilcoxon rank-sum and Mann–Whitney tests were used as appropriate for comparison of continuous variables, and the Chi-squared test was used for comparison of categorical variables. Pearson and Spearman’s correlation analysis was used to detect correlation. Multivariable logistic regression analyses were performed to test the independent strengths of associations*.* Statistical analyses were conducted with GraphPad Prism^®^ 8 and STATA^®^. For microRNAs, analysis was carried out in GraphPad Prism^®^ six by unpaired t-test with Welch’s correction. Data were normalized to endogenous control miR-191 and are presented as mean ± SEM. A two-tailed p-value < 0.05 was considered significant for all studies. The present study represents a pilot study and as the expected small effect size, the aim of the study was to recruit at least 25 subjects each group [28].

## Results

**Fig. 1:**
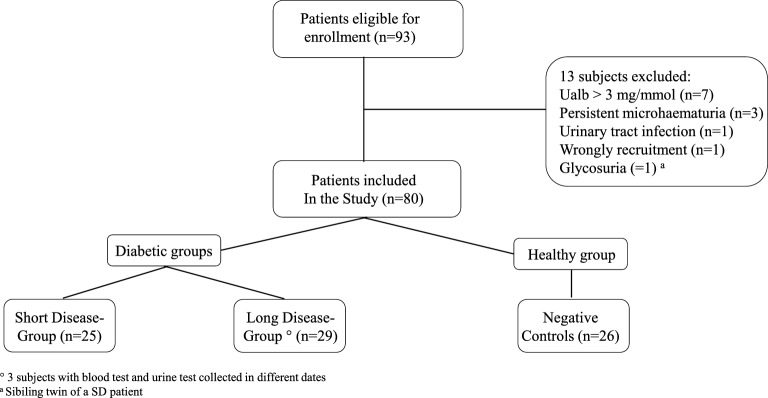
Patient recruitment and distribution into the different study groups

Over one year, 93 subjects were screened for the study, of whom 80 were recruited and included in the final analysis. Thirteen patients were excluded as they met one or more exclusion criteria. The distribution of the patients across the three groups and the exclusion criteria for each patient are shown in Fig. 1.

### Baseline features

Baseline demographic and clinical features of the subjects are shown in Table 1 and Additional file 1: Table S1. The biochemical and metabolic characteristics of the diabetic children are shown in Additional file 1: Table S2.

**Table 1:** Baseline features of the study population

	Short duration group	Long duration group	Healthy group	p value
Age (years)	11.8 (6.6, 12.6)	16.7 (14.7, 18.0)	11.0 (9.4, 13.4)	< 0.0001^a^
Age at T1DM onset (years)	9.7 (4.5, 12.4)	3.3 (2.4, 5.1)	–	< 0.0001
Duration of T1DM (years)	1.9 (1.1, 2.1)	12.4 (11.6, 14.0)	–	< 0.0001
HbA1c^b^ (mmol/mol)	49 (52, 59)	60 (51, 67)	–	0.03
Serum creatinine (mg/dL)	0.51 ± 0.15	0.71 ± 0.13	–	< 0.0001
eGFR (mL/min/1.73 m^2^)^c^ [25]	160 ± 26	145 ± 21	–	0.006

^a^Significantly higher in LD group compared to both SD and H groups

^b^Normal range (20–42 mmol/mol)

^c^Estimated glomerular filtration rate (eGFR) is calculated using the Schwartz formula for children and young adults between the age of 1 and 19. Creatinine is dependent on muscle mass and, as expected, was higher in adolescents with long-standing T1DM compared with the younger counterpart of children and adolescents with short-duration disease. Accordingly, eGFR was lower in the LD-group. However, none of the subjects had impaired kidney function defined as a decrease of eGFR [29].

### Principal biomarkers

**Fig. 2:**
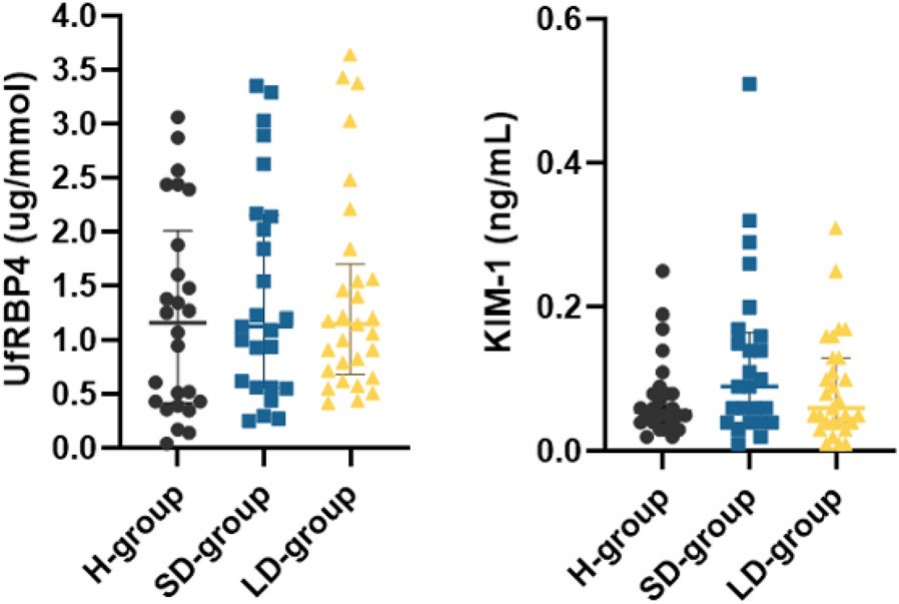
UfRBP4 and KIM-1 across the study groups. Both biomarkers were not significantly different across children with or without T1DM

The first aim of the study was to establish whether a functional tubular biomarker such as UfRBP4 and a tubular injury biomarker such as KIM-1 were significantly different between patients with a short duration of T1DM and healthy participants without DM, and between short and long duration T1DM patients. We found that both biomarkers were not significantly different across all paediatric groups (Table 2; Fig. 2).

**Table 2:** Principal biomarkers in the study population

	Short disease group (n = 25)	Long disease group (n = 29)	Healthy group (n = 26)	p-value
UfRBP4 (ug/mmol)	1.13 (0.6, 2.1)	1.15 (0.7, 1.6)	1.16 (0.43, 1.81)	0.7
Ualb (mg/mmol)	0.6 (0.4, 0.8)	0.4 (0.4, 0.9)	0.7 (0.4, 1.0)	0.5
KIM-1 (ng/mL)	0.09 (0.04,0.15)	0.06 (0.04,0.13)	0.06 (0.04,0.08)	0.07

The three biomarkers are expressed in relation to urine creatinine

*UfRBP4* urine free retinol-binding protein 4 to urine creatinine; *Ualb* urine albumin to urine creatinine; *KIM-1* kidney injury molecule 1 to urine creatinine

**Fig. 3:**
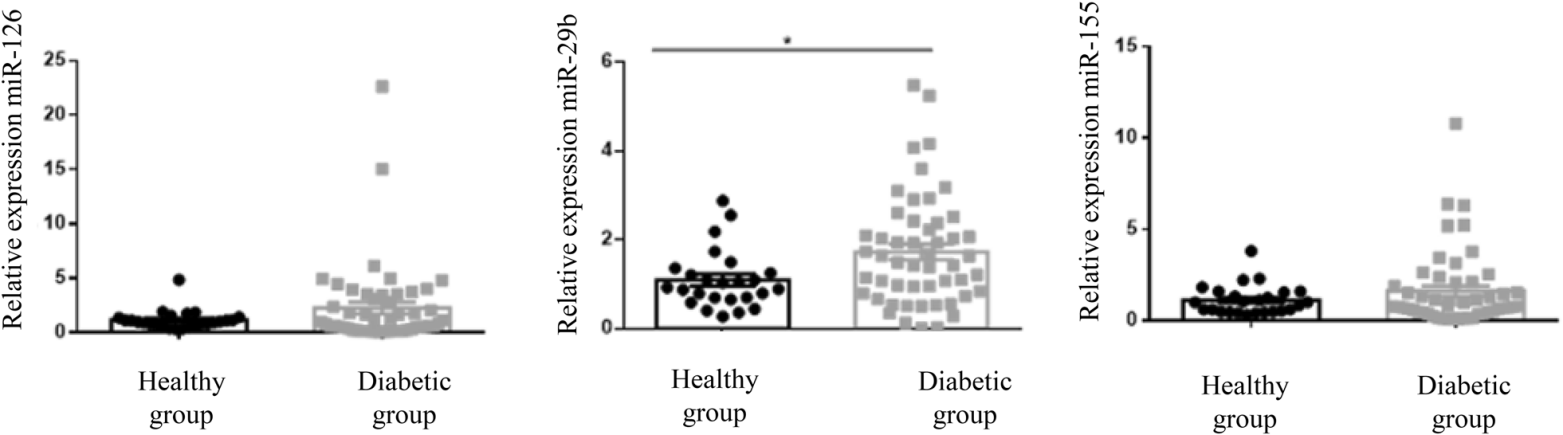
Expression of miR-126, miR-29b and miR-155 in healthy children compared to diabetic children (SD and LD groups). miR-29b was higher in diabetic children overall compared to controls (*p = 0.03), whereas miR-155 and miR-126 were similar across the two groups

On average, all three groups had normal and not significantly different Ualb. Among microRNAs, miR-29b was higher in diabetic children overall compared with healthy controls (p = 0.03), whereas miR-155 and miR-126 were not significantly different (Fig. 3). No differences were found between the SD and LD group in expression of the three microRNAs. No associations were identified between urinary excretion of the microRNAs and sex, age, eGFR, Ualb and glycaemic control as determined by HbA1c measurement.

**Fig. 4:**
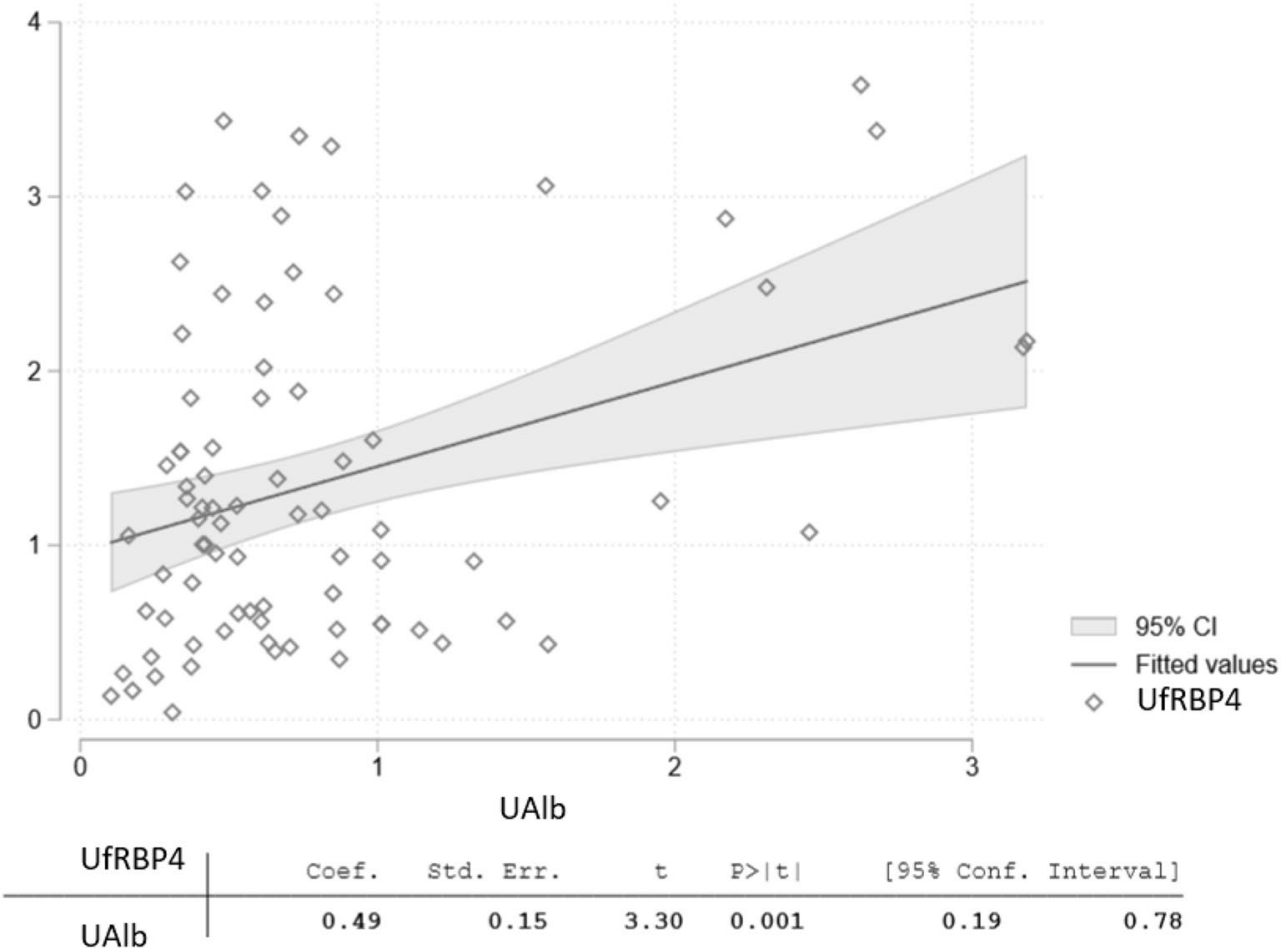
Relationship between UfRBP4 and Ualb across all study groups. *UfRBP4* urine free retinol-binding protein 4 (µg/mmol); *Ualb* urinary albumin expressed as albumin to creatinine ratio (mg/mmol)

UfRBP4, Ualb and KIM-1 showed no significant differences by sex, metabolic control of T1DM or T1DM-related complications requiring admission to hospital (Table 3). UfRBP4 was correlated with Ualb in the whole population and in each group of the study (Fig. 4), whereas KIM-1 did not correlate with either UfRBP4 or UAlb (Additional file 1: Fig. S1).

**Table 3:** Comparison between the different subgroups for UfRBP4, Ualb and KIM-1

	Male (n = 46)	Female (n = 34)	*p*	HbA1c target ≤ 59 mmol/mol (n = 35)	HbA1c target > 59 mmol/mol (n = 19)	*p*	T1DM- complications (n = 16)	No T1DM-Complications (n = 38)	*p*	Hyper-filtration (n = 11)	No Hyper-filtration (n = 40)	*p*
UfRBP4 *(µg/mmol)*	1.1 (0.5, 1.5)	1.1 (0.6, 2.4)	0.3	1.2 (0.8, 2.0)	1.1 (0.6, 1.6)	0.3	1.1 (0.7, 1.8)	1.2 (0.6, 1.9)	0.9	1.0 (0.6, 1.6)	1.2 (0.7, 1.2)	0.3
Ualb *(mg/mmol)*	0.5 (0.4, 0.9)	0.7 (0.4, 1.4)	0.1	0.6 (0.4, 0.8)	0.4 (0.3, 1.0)	0.6	0.7 ± 0.5	0.8 ± 0.6	0.7	0.7 ± 0.6	0.9 ± 0.7	0.3
KIM-1 *(ng/mL)*	0.07 (0.04,0.11)	0.06 (0.04,0.15)	0.7	0.06 (0.04,0.11)	0.13 (0.05,0.15)	0.1	0.06 (0.04,0.12)	0.06 (0.04,0.10)	0.7	0.07 (0.05,0.16)	0.08 (0.04,0.13)	0.5

^a^Definition of Hyperfiltration: eGFR that exceeds two standard deviations above mean GFR of an age-matched normal (i.e., without evidence of renal disease) population, where glomerular filtration rate was determined by inulin clearance [26]. Notably, the prevalence of hyperfiltration was not significantly different between SD and LD-group (p = 0.8)

^p = p-value^

## Discussion

In our short-disease (SD) cohort, UfRBP4 was not different from age-matched healthy controls (H) or from children with longer duration (LD) diabetes. The process underlying increased urinary excretion of fRBP4 appears relatively late in the course of T1DM, with studies of URBP4 indicating that increases in this protein are relatively common in late stage T1 and T2DM in adults [9, 21, 25]. The current explanation for increased excretion of URBP4 is tubular dysfunction and disease. However, with predominant tubular damage, as in the renal Fanconi syndrome, the increase in fRBP4 is several orders of magnitude higher [30]. The absence of even a mild increase in UfRBP4 probably excludes an early tubular abnormality in T1DM, at least one that affects tubular protein handling. A recent model of glomerular filtration and tubular absorption of RBP4 by Edwards et al*.* suggests that, if proximal tubular reabsorption is intact, measurement of urine RBP4 is not superior to albumin in detecting increases in glomerular permeability [31]. The clinical results here are consistent with this prediction.

KIM-1 is a non-invasive kidney biomarker that has shown reliable performance in identifying children with chronic kidney disease at risk of progression [32]. In T1DM, KIM-1 was overexpressed very early in the course of the disease with a predictive value of progression of DKD [13], and was linked to reduced kidney function in a GWAS study [33]. Recently, the association of endocytic function of KIM-1 on epithelial tubular cells of PT and pathogenesis of DKD has been reported, making inhibition of KIM-1 a potentially attractive therapeutic strategy to prevent progression of DKD [12]. Like UfRBP4, KIM-1 showed no elevation in children with short duration DM compared with healthy counterparts, as well as no overexpression in the LD group. KIM-1, a validated kidney injury biomarker in DKD and in children did not detect signs of kidney damage in our study population.

We also explored the expression of three different microRNAs reported previously to show a microRNA signature associated with DKD [15]. One of the advantages of using non-invasive biomarkers like microRNAs is their stability in many biological fluids. Urine is an unstable *milieu* in which many proteins undergo degradation. MicroRNAs in body fluids may be evaluated in total or following subfractionation, for example of exosomes and other microvesicles. Our previous work has demonstrated that in urine, microRNAs are stabilised by presence in microvesicles and also by association with RNA binding proteins, including Ago proteins, while non-protected microRNAs are rapidly degraded [27]. Future work to additionally characterise microRNA localisation within microvesicles versus stabilisation by proteins may help determine cellular source, helping to uncover microRNA-directed regulatory mechanisms. Urinary expression of miR-155 and miR-126 was similar across the three paediatric groups, whereas miR-29b was more highly expressed in the pooled diabetic group compared with healthy controls. Although no differences were found when the SD group was compared with the LD group, miR-29b may still provide an early signal for DKD, since it has been found to be enriched in glomerular endothelial cells and released from these cells in response to DKD-linked cytokines [15]. miR-155 and miR-126 were found to be elevated in cohorts with established DKD in other studies, but we did not find this in early stages of T1DM in our carefully phenotyped patients with normal renal function and no microalbuminuria. These findings are also consistent with our UfRBP4 and KIM-1 results in detecting no sign of early renal involvement in T1DM patients compared with controls. Recent improvements in the treatment of T1DM have resulted in better metabolic control, which will decrease the incidence of complications of T1DM, including nephropathy [34, 35].

A surprising finding was the lack of any increase in biomarkers in the LD group. The absence of any difference between LD and healthy controls suggests that our negative findings indicate the absence of nephropathy in this group of young T1DM patients, regardless of the duration of diabetes. The susceptibility to DKD reflects genetically and epigenetically determined differences in the kidney response to metabolic and autoimmune disease [36] that are magnified in the presence of hyperglycemia [37]. In patients with T1 and T2 diabetes, a cluster of insulin-resistant individuals has been found to have a significantly higher risk of developing DKD [38]. As already mentioned, the absence of nephropathy in our cohort of children may reflect optimal clinical management and glycaemic control [34]. In children, more intensive insulin regimens are recommended, because they are more likely to reach and maintain glycated haemoglobin (HbA1C) targets with better clinical outcomes [39].

Our study has two main limitations. We could not evaluate routine blood chemistry in healthy children because of ethical restrictions. Moreover, the controls were all siblings of patients with T1DM and their lifetime risk of developing DM is known to be increased relative to the general population [40]. However, the controls did have a random fasting glucose measurement within six months of urine collection.

The current literature describes very few studies evaluating a panel of urinary biomarkers in diabetic children and adolescents, and none has recruited patients with T1DM of short duration to compare with those of longer duration. Study of T1DM does make interpretation of our results easier, because of the known time of onset of diabetes and the absence of any potentially confounding co-morbidities in otherwise healthy children, in contrast with T2DM.

Taken together, UfRBP4, KIM-1, miR-155 and miR-126 were not increased in young T1DM patients with normal renal function and no urinary albumin excretion. These biomarkers have been reported to be elevated in cohorts with established DKD, mainly in adults, in other studies; however, we have not found them to be increased in carefully phenotyped children, adolescents and young adults with T1DM, and they are unlikely to be of added value in screening for early DKD in the absence of albuminuria.

## Summary conclusions

A panel of biomarkers, including functional and structural biomarkers such as UfRBP4 and KIM-1, and three tested microRNAs linked previously to DKD in an adult setting showed no differences in children, adolescents, and young adults with different durations of T1DM and without other evidence of nephropathy. These findings support the specificity of the biomarkers tested for those diabetics with nephropathy. Further studies are required to determine whether very early nephropathy is discernible using these biomarkers. Our findings also suggest that children with T1DM today have a more favourable renal outlook, probably the result of improvements in early diagnosis and routine clinical care.

## Supplementary Information

**Additional file 1**: **TableS1 **Baseline features of continuous variables of the study population. **Table S2 **Baseline biochemical and metabolic parameters of the diabetic population. **Table S3 **Bivariate correlations of several variables in the study population. **Fig.S1 **Relationship between KIM-1 with UfRBP4 and Ualb across all study groups. KIM-1: Kidney injury molecule 1 (ng/mL); UfRBP4: urine free retinol-binding protein 4 (ug/mmol); Ualb: urinary albumin expressed as albumin to creatinine ratio (mg/mmol).

## Data Availability

The datasets used and/or analysed during the current study are available from the corresponding author on reasonable request.

## Abbreviations

DKD: Diabetic kidney disease
TQDM: Type1 diabetes mellitus
UfRBP4: Urine free retinol-binding protein 4
UAlb: Albumin
KIM-1: Kidney injury molecule-1
SD: Diabetes of short-duration
LD: Diabetes of long-duration
H: Non-diabetic siblings
LMWP: Low molecular weight protein
CAKUT: Congenital anomalies of the kidney and urinary tract
eGFR: Estimated glomerular filtration rate
