# Supplementary material for: Exploration of a panel of urine biomarkers of kidney disease in two paediatric cohorts with Type 1 diabetes mellitus of differing duration
Source: Diabetol Metab Syndr. 2022 May 12;14:71. doi: 10.1186/s13098-022-00839-4 (PMC9097324; doi:10.1186/s13098-022-00839-4)
Supplement: Supplementary file 1 — Additional file 1: TableS1 Baseline features of continuous variables of the study population. Table S2 Baseline biochemical and metabolic parameters of the diabetic population. Table S3 Bivariate correlations of several variables in the study population. Fig.S1 Relationship between KIM-1 with UfRBP4 and Ualb across all study groups. KIM-1: Kidney injury molecule 1 (ng/mL); UfRBP4: urine free retinol-binding protein 4 (ug/mmol); Ualb: urinary albumin expressed as albumin to creatinine ratio (mg/mmol). [file 13098_2022_839_MOESM1_ESM.pdf]

SUPPLEMENTARY MATERIALS

Table S1 Baseline features of continuous variables of the study population

Parameters	Short Disease Group (25 subjects)	Long Disease Group (29 subjects)	Healthy Group (26 subjects)	p-value
Male sex	52% (n=13)	62% (n=18)	58% (n=15)	0.78
Hyperfiltration	50% (n=12)	43% (n=12)	-	0.82
Antithyroid Ab positivity	24% (n=6)	17% (n=5)	-	0.40
Antitransglutaminase Ab positivity	16% (n=4)	7% (n=2)	4% (n=1)	0.73
Good metabolic control (HbA1c target <59 mmol/mol) [a]	72% (n=18)	59% (n=17)	-	0.39

[a]According to the guidelines from the American Diabetes Association (ADA) and the International Society for Paediatric and Adolescent Diabetes (ISPAD)

Table S2 Baseline biochemical and metabolic parameters of the diabetic population

Parameters (normal range & unit of measurement)	Short Disease Group	Long Disease Group	p Value
**Fasting glucose level (90-130 mg/dL) [a]**	158 ± 53	179 ± 77	0.6
**HbA1c (20-42 mmol/mol) [b]**	55 ± 10	61 ± 11	0.03
**Uric acid (2.5-5.4 mg/dL)**	3.2 ± 1.1	4.0 ± 1.3	0.004
**Serum creatinine (0.5-0.9 mg/dL)**	0.5 ± 0.1	0.7 ± 0.1	<0.0001
**eGFR (xx mL/min/1.73 m^2) [c]**	160 ± 26	145 ± 21	0.006
**Total cholesterol (120-180 mg/dL)**	163 ± 25	159 ± 31	0.65
**Triglycerides (<150 mg/dL)**	50 (41, 56)	68 (56, 85)	0.02
**High-density lipoprotein (>46 mg/dL)**	66 ± 14	64 ± 17	0.59
**Aspartate transaminase (9-31 U/L)**	20 (12, 25)	16 (11, 20)	0.16
**Alanine transaminase (11-29 U/L)**	17 (15, 20)	19 (15, 23)	0.32
**Gamma-glutamyltransferase (10-38 U/L)**	14 (13, 16)	16 (12, 19)	0.07
**Thyroid-stimulating hormone (0.3-4.2 mIU/L)**	2.5 ± 1.3	2.3 ± 0.8	0.43

[a] target blood glucose levels before meals according to American Diabetes Association (ADA) and the International Society for Paediatric and Adolescent Diabetes (ISPAD).

[b] target values better explained in section 'Methods'

[c] definition of normal GFR in footnote of table1

Table S3 Bivariate correlations of several variables in the study population

	UfRBP4 (µg/mmol)	
	Correlation coefficient (r)	p-value
**BMI**	0.007	0.9
**GLC**	−0.2	0.07
**SBP**	0.157	0.3
**DBP**	−0.05	0.70
**HbA1c**	−0.16	0.5
**DM Duration**	−0.03	0.8
**Age**	0.0007	0.9
**pH**	−0.06	0.15
**PS**	−0.07	0.07
**eGFR**	0.03	0.6
**Creatinine**	0.04	0.7
**Cholesterol**	0.13	0.31
**HDL**	0.14	0.30
**Triglycerides**	−0.05	0.70
**Insulin dose**	0.029	0.83
**Uric acid**	−0.11	0.40

r, Pearson correlation coefficient or nonparametric Spearman correlation coefficient

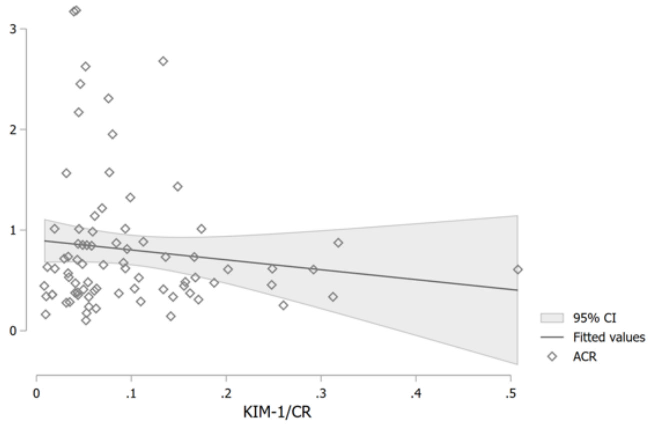

acr	Coef.	Std. Err.	t	P>|t|	[95% Conf.	Interval]
kim1cr	−0.981	0.891	−1.10	0.274	−2.754	0.792
_cons	0.901	0.115	7.84	0.000	0.672	1.129

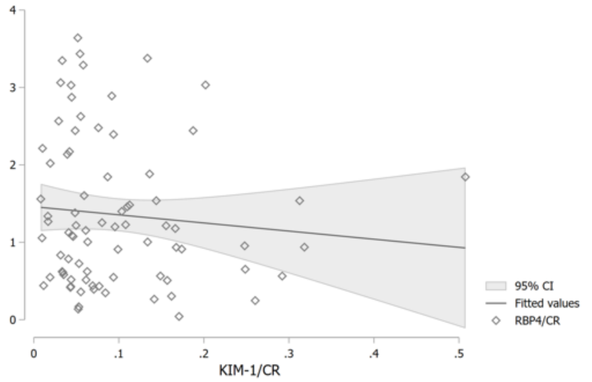

rbp4cr	Coef.	Std. Err.	t	P>|t|	[95% Conf.	Interval]
kim1cr	−1.050	1.242	−0.84	0.401	−3.523	1.424
_cons	1.460	0.160	9.11	0.000	1.141	1.778

Fig. S1 Relationship between KIM-1 with UfRBP4 and Ualb across all study groups. KIM-1: Kidney injury molecule 1 (ng/mL); UfRBP4: urine free retinol-binding protein 4 (ug/mmol); Ualb: urinary albumin expressed as albumin to creatinine ratio (mg/mmol).
